# ESR Essentials: artificial intelligence in breast imaging—practice recommendations by the European Society of Breast Imaging

**DOI:** 10.1007/s00330-025-11954-x

**Published:** 2025-08-26

**Authors:** Simone Schiaffino, Daniela Bernardi, Nuala Healy, Maria Adele Marino, Valeria Romeo, Ioannis Sechopoulos, Ritse M. Mann, Katja Pinker

**Affiliations:** 1 Imaging Institute of Southern Switzerland (IIMSI), Ente Ospedaliero Cantonale (EOC), Lugano, Switzerland; 2 Faculty of Biomedical Sciences, Università della Svizzera Italiana, Lugano, Switzerland; 3 IRCCS Humanitas Research Hospital, Milan, Italy; 4 Department of Biomedical Sciences, Humanitas University, Milan, Italy; 5 Beaumont Breast Centre, Beaumont Hospital, Dublin, Ireland; 6 Department of Radiology, Royal College of Surgeons, Dublin, Ireland; 7 Department of Biomedical Sciences and Morphologic and Functional Imaging, University of Messina, Messina, Italy; 8 Department of Advanced Biomedical Sciences, University of Naples Federico II, Naples, Italy; 9 Department of Diagnostic Imaging, Radboud University Medical Center, Nijmegen, The Netherlands; 10 Department of Radiology, Netherlands Cancer Institute, Amsterdam, The Netherlands; 11 Department of Radiology, Columbia University, Vagelos College of Physicians and Surgeons, New York, NY US

**Keywords:** Breast neoplasms, Artificial intelligence, Mammography, Magnetic resonance imaging, Large language models

## Abstract

**Abstract:**

Artificial intelligence (AI) can enhance the diagnostic performance of breast cancer imaging and improve workflow optimization, potentially mitigating excessive radiologist workload and suboptimal diagnostic accuracy. AI can also boost imaging capabilities through individual risk prediction, molecular subtyping, and neoadjuvant therapy response predictions. Evidence demonstrates AI’s potential across multiple modalities. The most robust data come from mammographic screening, where AI models improve diagnostic accuracy and optimize workflow, but rigorous post-market surveillance is required before any implementation strategy in this field. Commercial tools for digital breast tomosynthesis and ultrasound, potentially able to reduce interpretation time and improve accuracy, are also available, but post-implementation evaluation studies are likewise lacking. Besides basic tools for breast MRI with limited proven clinical benefit, AI applications for other modalities are not yet commercially available. Applications in contrast-enhanced mammography are still in the research stage, especially for radiomics-based molecular subtype classification. Applications of Large Language Models (LLMs) are in their infancy, and there are currently no clinical applications. Consequently, and despite their promise, all commercially available AI tools for breast imaging should currently still be regarded as techniques that, at best, aid radiologists in image evaluation. Their use is therefore optional, and the findings may always be overruled.

**Key Points:**

- *AI systems improve diagnostic accuracy and efficiency of mammography screening, but long-term outcomes data are lacking.*
- *Commercial tools for digital breast tomosynthesis and ultrasound are available, but post-implementation evaluation studies are lacking.*
- *AI tools for breast imaging should still be regarded as a non-obligatory aid to radiologists for image interpretation.*

## Key recommendations

- Implementing AI systems in mammography screening seems to increase diagnostic accuracy and efficiency. However, for any implementation strategy rigorous post-market surveillance is mandatory (Level of evidence: Moderate).
- Commercial tools for digital breast tomosynthesis, able to reduce interpretation time and improve accuracy, and ultrasound, aiding lesion classification and reducing inter-reader variability, can be used, although their actual impact in clinical practice is not well evaluated (Level of evidence: Moderate).
- The current use of AI tools in clinical breast imaging should be considered an aid for the reporting radiologist. Due to the absence of evidence of improved long-term clinical outcomes with AI, findings may be accepted or overruled by the radiologist taking all clinical information in consideration without need for explanation in the report (Level of evidence: Moderate).

## Introduction

Artificial intelligence (AI) can have a positive impact on breast cancer imaging by filling its main gaps on the one side, such as excessive radiologists’ workload and limited diagnostic accuracy, and by boosting its capabilities on the other (Table 1). AI systems can enhance traditional imaging performance mainly by improving lesion detection, specificity, and workflow efficiency, but despite the high potential, their implementation requires robust governance structures to ensure safety, quality, and efficiency. Moreover, cost-effectiveness and large-scale validation studies are still lacking.

**Table 1:** Summary of the main available evidence and future perspectives for each breast imaging modality

Imaging modality	Main available evidence	Future perspectives
Screening digital mammography	• Enhanced diagnostic performance, even in prospective randomized studies • Significant workload reduction • Better risk assessment than traditional models	• Extensive external validation studies • Assessment of potential impact on long-term outcomes • Post-marketing surveillance
Digital breast tomosynthesis	• Significant reading time reduction • Optimized workflows • High accuracy, especially in dense breasts	• Validation on large-scale prospective studies • Assessment of potential impact on long-term outcomes • Further integration across vendor systems
Breast ultrasound	• Improved lesion characterization • Reduce unnecessary biopsies • Integration with elastography • Good performance in axillary staging	• Standardize guidelines • Triaging in resource-limited environments • Neoadjuvant therapy response prediction
Breast MRI screening	• Limited value of commercial CAD tools • Preliminary studies on screening sets • Limited diagnostic performance in real screening cohorts	• Validation in real screening cohorts • Workload reduction
Breast MRI advanced applications	• Promising results in recurrence scores and risk prediction • Good performance on molecular subtyping prediction • Improved neoadjuvant therapy response prediction	• Personalized screening based on risk prediction • Neoadjuvant treatment tuning • Development of contrast-free imaging
Contrast-enhanced mammography	• Radiomics-based molecular subtype classification • Promising synthetic image generation	• External validation studies • Development of reduced-contrast or contrast-free techniques

In mammography, several commercial tools are available, but their impact on long-term outcomes has not been assessed yet. Commercial tools for digital breast tomosynthesis (DBT) are able to reduce interpretation time and improve accuracy, and for ultrasound (US), they can aid in lesion classification and reduce inter-reader variability, but these lack post-implementation evaluation studies. In breast MRI, commercial computer-aided diagnosis (CAD) tools have existed for decades, yet offer limited added value for experienced radiologists. Meanwhile, advanced AI applications, including molecular subtype and risk prediction, and treatment response assessment, show promising results but are still largely confined to the research setting.

**Fig. 1:**
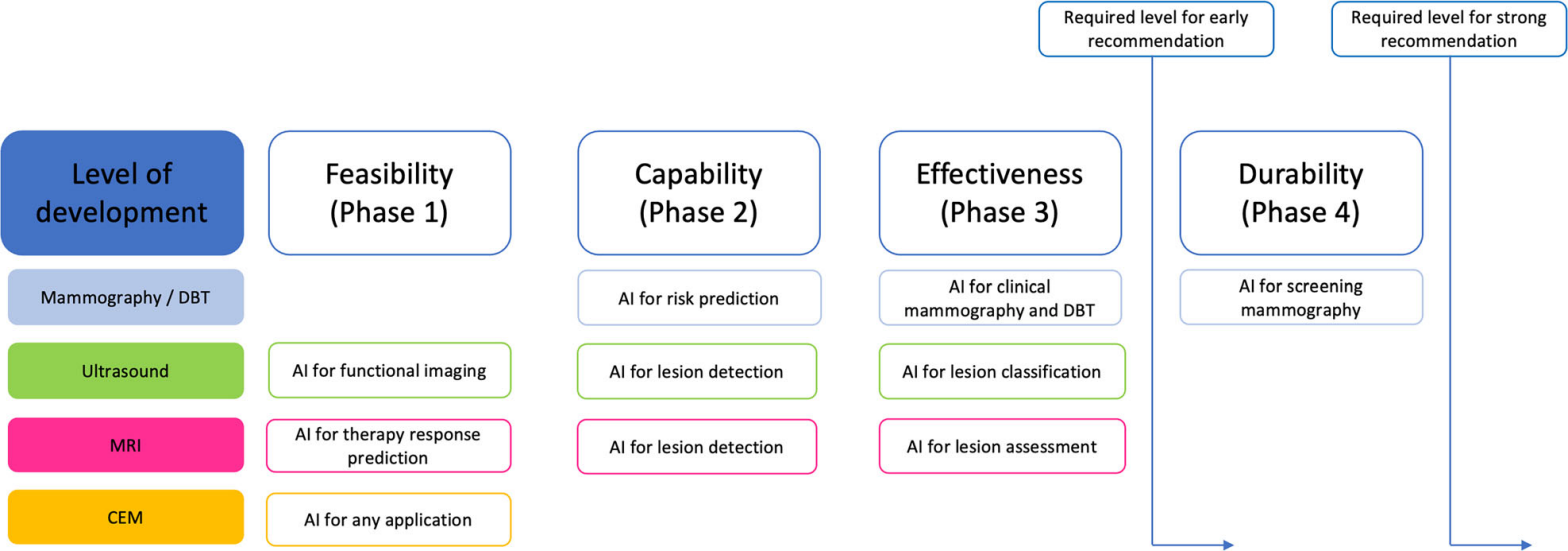
The World Health Organization describes 4 different phases for generating evidence for artificial intelligence-based medical devices (https://iris.who.int/). This runs from feasibility (phase 1) to durability (phase 4), the last phase requiring continuous post-market surveillance of AI performance. We feel that strong recommendations can only be made when AI tools achieve this. This flowchart shows the status of artificial intelligence algorithms in the different imaging modalities

The World Health Organization classifies the evidence for artificial intelligence-based medical devices into four phases (https://iris.who.int/), from feasibility (phase 1) to durability (phase 4), with the last phase requiring continuous post-market surveillance of AI performance (Fig. 1). As AI models evolve, radiologists should assess their utility and ensure implementation is based on robust evidence; strong recommendations should follow when AI tools reach the last phase.

## AI applications in mammographic screening

**Fig. 2:**
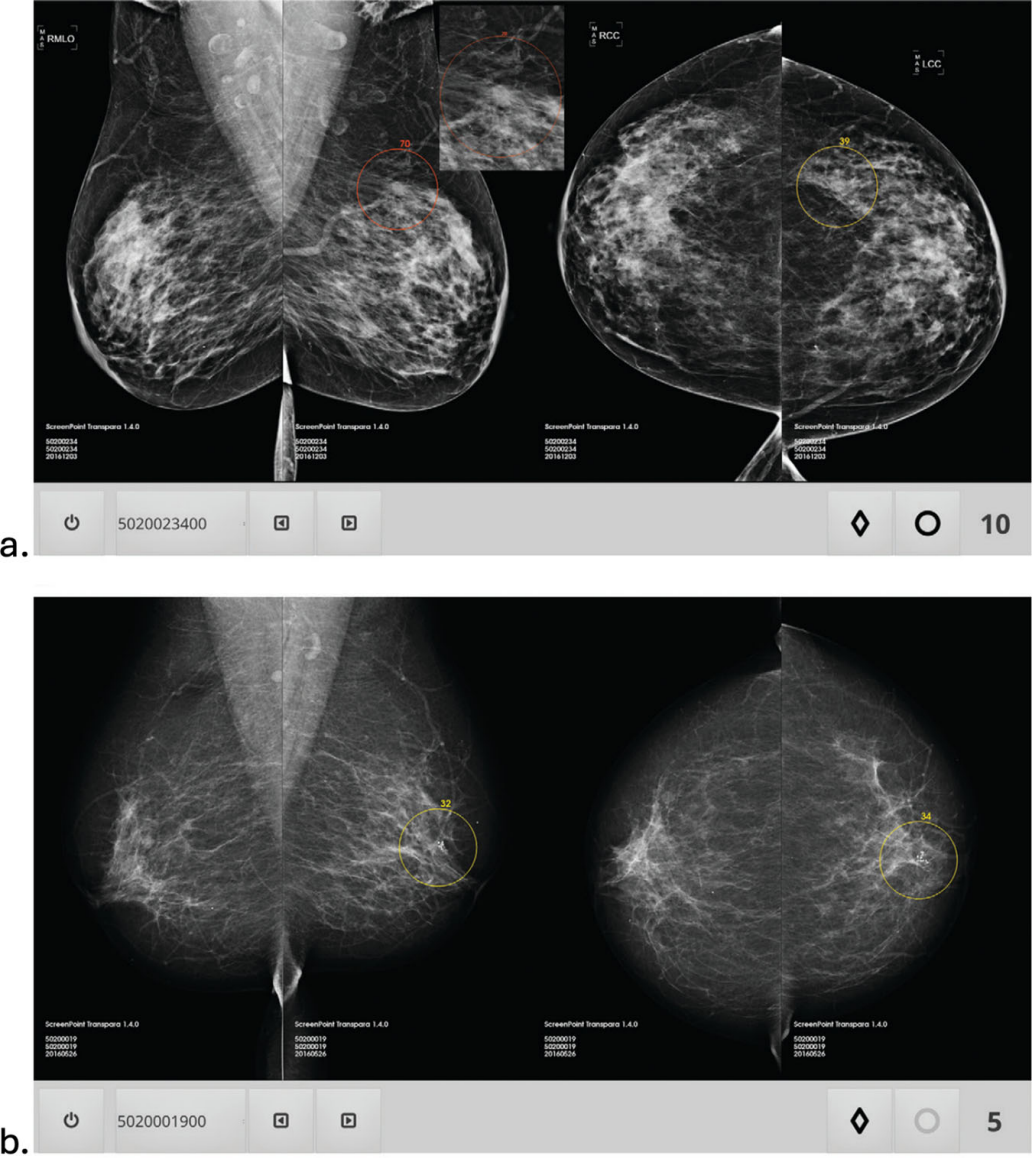
**a** Mammograms in a 71-year-old woman with invasive ductal carcinoma (outlined and with level of suspicion score assigned by computer system). Patient was recalled (Breast Imaging Reporting and Data System (BI-RADS) score, ≥ 3) by four of 14 radiologists when reading unaided and by 11 of 14 radiologists using artificial intelligence (AI) system for support. Outlined areas and scores are shown as in viewer of AI system. **b** Mammograms in a 62-year-old woman without cancer, who was recalled (BI-RADS score, ≥ 3) by 12 of 14 radiologists when reading unaided and by seven of 14 readers when using AI system for support. Outlined areas and scores are shown as in viewer of AI system

AI is emerging as a powerful tool in breast cancer screening by improving digital mammography (DM) accuracy and efficiency [1, 2], and several commercially systems are today available (Fig. 2). As an example, in a multireader multicase setting one was able to increase, compared to standard reading, AUC from 0.87 to 0.89, sensitivity from 83 to 86% and specificity from 77 to 79%, with no increase in reading time (146 s vs. 149 s) [3]. The same model outperformed 61% of 101 radiologists across nine datasets from prior multireader, multicase studies as a standalone reader, even without any access to prior mammograms [4]. A systematic review including sixteen studies validated AI’s performance across DM and DBT [2]. AI generally outperformed radiologists in sensitivity but with slightly lower specificity. In reader studies, the AI pooled AUC was significantly higher when applied to DM (0.87 vs. 0.81) or DBT (0.90 vs. 0.79) compared to human readers. However, AI performance was similar to human assessment when compared to historical reads of consecutive screening cohorts. While promising, the limited number of DBT studies and variability in study designs warranted further research.

AI integration into screening workflows has shown significant benefits. In general, screening workflows include different reading strategies in programs worldwide, including single reading or double reading (independent or consensus). McKinney et al demonstrated that AI as a second reader reduced radiologist workload by 88% while maintaining non-inferior accuracy [5]. The decision-referral model further enhances AI’s utility by triaging normal and suspicious cases for human review. A German study [6] with over one million mammograms compared AI as a standalone system, AI within a decision-referral framework, and unaided human readers. In standalone mode, despite strong performance (AUC 0.95, external validation), the AI system was less accurate than unaided radiologists. The decision-referral approach demonstrated marked improvements over unaided radiologists, with reduced workload. Notably, subgroup analyses showed AI improved the detection of invasive lesions, small tumors, and in dense breast.

Evidence from early prospective studies within a population-based screening program suggests AI’s feasibility and efficiency. A prospective study in Hungary explored the implementation of AI as an additional reader, improving early cancer detection with minimal increase in the recall rate. AI-assisted workflows, with flagged cases not recalled by standard double reading, achieved a 5–13% relative increase in cancer detection rate, with a modest increase in recall rate [7]. The MASAI trial [1, 8], a large-scale randomized controlled study, significantly boosted the available evidence, evaluating AI-supported mammography screening against standard double reading in Sweden (unblinded double reading, the standard of care in the regional screening program in the Skåne region). AI’s triage system stratified cases, directing low-risk to single reading and high-risk to double reading. AI-assisted screening demonstrated non-inferior cancer detection rates (6.1 vs. 5.1 per 1000 participants) while reducing the radiologists’ screen-reading workload by 44%. ScreenTrustCAD, another prospective Swedish study, compared traditional double reading (double reading plus consensus) with alternative strategies, including hybrid double reading (AI and human), standalone AI, and triple reading (two radiologists plus AI). The hybrid double reading detected 1.04 times more cancers while reducing workload by 44%, compared to the traditional approach. Standalone AI achieved non-inferior results but did not surpass traditional double reading, while the triple-reading strategy, despite the highest detection rate, raised concerns about its efficiency [9]. Long-term outcomes from these trials are not yet published at the time of writing.

In screening, AI models can also provide significant support in risk assessment, toward personalized screening. A study compared a mammogram-based deep learning (DL) risk assessment model with traditional risk models (Tyrer-Cuzick and BCRAT) for identifying high-risk patients undergoing breast MRI screening. The model showed a significantly higher cancer detection rate (20.6 per 1000) than traditional models (6.0–6.8 per 1000) [10]. In a recent study, radiomic parenchymal phenotypes extracted from DM were significantly associated with increased risk of invasive breast cancer, especially in cases with false-negative findings and symptomatic interval cancers, across both Black and White women, independent of traditional breast density measures [11].

These findings suggest that DL- and radiomic-based risk assessment could enhance breast cancer screening by more accurately identifying individual risk.

Based on current evidence, implementing AI in mammographic screening is likely beneficial and could be carefully recommended. It has the potential to at least partly replace one of the readers in a double reading setting. However, the optimal way of implementing AI is unknown. Any approach to implementation should therefore be accompanied by rigorous post-market surveillance and monitoring of relevant outcome parameters such as recall rates, biopsy rates, and cancer detection rates.

## The role of AI tools in digital breast tomosynthesis

**Fig. 3:**
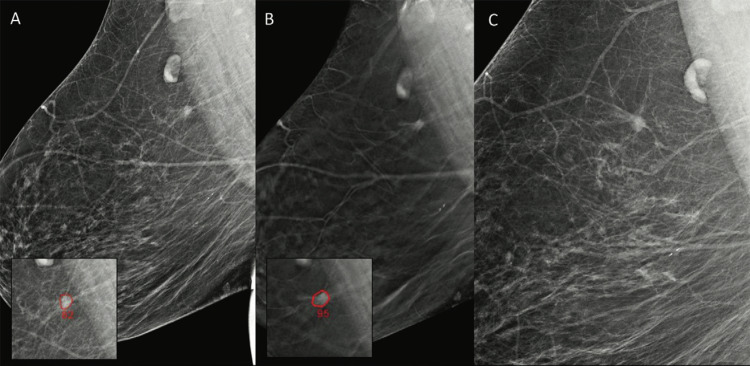
A 66-year-old woman underwent screening mammography without any recall on the original readings. A spiculated mass in the upper quadrants of the right breast was detected by AI (outlined) on DM (**A**) and DBT images (**B**). In **C**, DM image obtained 4 months later, after a palpable lump was discovered by the patient (not related to the actual cancer). Biopsy was performed, and an interval cancer, a 6 mm grade II invasive ductal carcinoma, was diagnosed in the lesion that would have been recalled by AI. Reproduced from [15] under the Creative Commons, CC BY 4.0 license

Commercially available systems have recently emerged with the capability to handle DBT across different vendors. While DBT diagnostic advantages over DM are clear, widespread adoption has been slowed by longer reading times and increased interpretative complexity [12]. Studies show that AI can significantly reduce reading times—by as much as 23–29%—while maintaining accuracy [13, 14]. By highlighting areas of interest and generating enhanced synthetic views, AI not only speeds up interpretation but also helps radiologists focus on key findings, ultimately improving efficiency. The algorithm used by Benedikt and colleagues in their study can select the suspicious lesion and extract it from the single tomosynthesis image, and blend it to the synthetic one, to increase the reading efficiency [14]. AI was also shown to be able to optimize screening workflows by triaging cases based on risk levels, reducing radiologists’ workload by 30% while increasing sensitivity by 17% (Fig. 3) [3, 15].

AI systems have achieved performances close to human double reading (AUC 0.93 vs. 0.98) [16, 17], particularly excelling in detecting abnormalities in dense breasts, where traditional methods often fall short [17].

Recently, AI-based CAD system developed for DM was applied to synthetic mammography [18]. AI-CAD showed lower sensitivity but higher specificity on synthetic images than DM, with comparable AUC. Results suggest that DM-based AI-CAD should not be applied to synthetic mammograms without proper tuning.

AI shows substantial promise in risk prediction by leveraging DL algorithms to extract and analyze imaging biomarkers, parenchymal texture, and mammographic breast density. These automated assessments enable stratification of individual cancer risk, potentially guiding personalized screening [19].

There is currently insufficient evidence for the recommendation of AI for DBT, but it may be used concurrently when interpreting tomosynthesis examinations as it may reduce reading times and potentially increase diagnostic accuracy.

## Ultrasound: from lesion classification to elastography

**Fig. 4:**
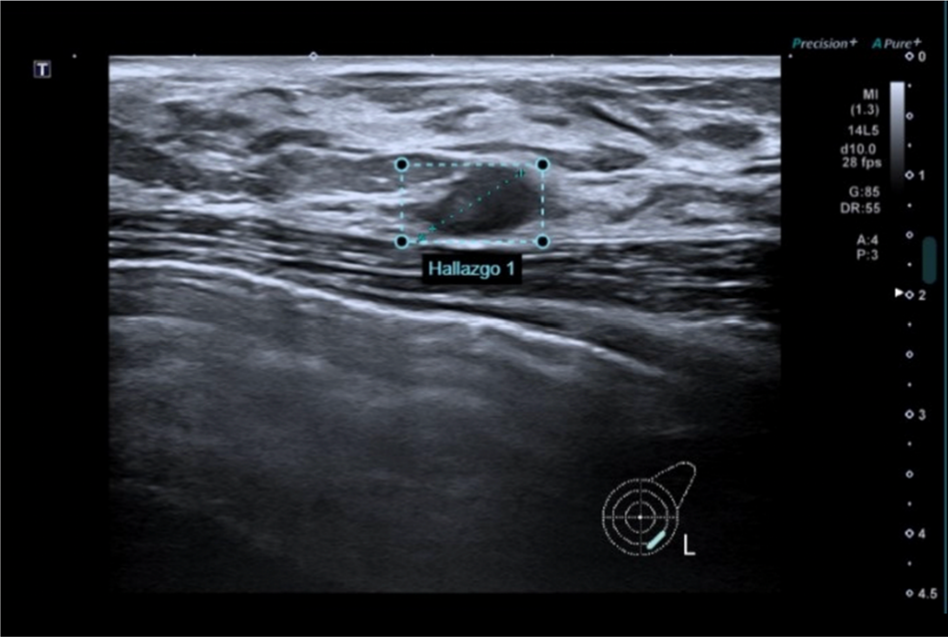
Suspicious ultrasound breast lesion rated as BI-RADS 4A using a commercially available AI software, confirmed at core-biopsy as invasive carcinoma. Reproduced from [21] under the Creative Commons, CC BY 4.0 license

AI applications have emerged as promising solutions to most of the breast US limitations, improving its lower specificity in women older than 50 and reducing image acquisition and review time [20, 21]. Improved breast lesions classification has been shown by several authors (Fig. 4). For example, Shen et al [22] demonstrated the value of computerized BI-RADS sonographic features assessment with an AUC of 0.97, with a potential improvement in the efficiency and significant reduction of unnecessary biopsies. Similarly, Niu et al [23], focusing on BI-RADS 4A lesions, identified US patterns able to differentiate benign tumors—such as internal calcifications and specific long axis angles—from malignant ones, characterized by margin lobulations and lower entropy. Further improvements came from DL advancements, particularly in convolutional neural networks (CNNs), with quicker and more accurate malignant lesions classification compared to previous architectures [24]. AI achieves diagnostic accuracy comparable to radiologists while offering a faster and more consistent learning curve, especially for less experienced readers [25]. Tools can exemplify AI’s integration into US devices, improving lesion characterization accuracy and reducing variability in BI-RADS classifications, promoting standardization [26].

Advanced US approaches, such as the shear wave elastography (SWE), combined with B-mode imaging, can improve lesion characterization by themselves [20], and even more with the combination with AI. La Rocca et al [27] developed a radiomics and machine learning approach combining B-mode US and SWE, outperforming radiologists using B-mode alone, with however limited impact of SWE images. Zhang et al [28] extended this approach with DL systems that automatically extract SWE features, with great performance (AUC 0.95).

Good performance was found in axillary staging too. Zhang et al [29] developed a computer-assisted model incorporating real-time elastography and B-mode US features with an AUC of 0.90 and 85% accuracy in depicting metastatic lymph nodes in breast cancer patients. Similar results were achieved in an US elastography radiomics nomogram for preoperative evaluation of the axillary lymph node (ALN) burden in early-stage breast cancer [30]. The authors found that SWE signature, US-reported ALN status, and molecular subtype were independent risk factors associated with ALN status, and the favorable predictive ability for ALN staging of the radiomic model could provide incremental information for decision-making in patients with early-stage breast cancer.

The integration of AI with automated breast ultrasound (ABUS) has demonstrated significant potential in enhancing breast cancer diagnosis, treatment evaluation, and the prediction of molecular markers. ABUS radiomics is increasingly being combined with histological grading, immunohistochemical markers, and clinical parameters such as survival outcomes and hematological profiles, enabling more comprehensive tumor characterization. Nevertheless, key challenges remain, including the development of DL-based multimodal data fusion techniques, robust data privacy protection mechanisms, and improved model interpretability [31].

Regulatory-approved AI systems for breast US do exist and may be used to better classify breast lesions and reduce biopsies for benign findings. However, post-implementation surveillance studies are lacking and should be conducted to assess the actual value of this use case [20]. Looking further into the future, AI’s applications in breast US may include triaging patients with detectable tumors in resource-limited environments and predicting responses to neoadjuvant chemotherapy.

## MRI: the research lab

**Fig. 5:**
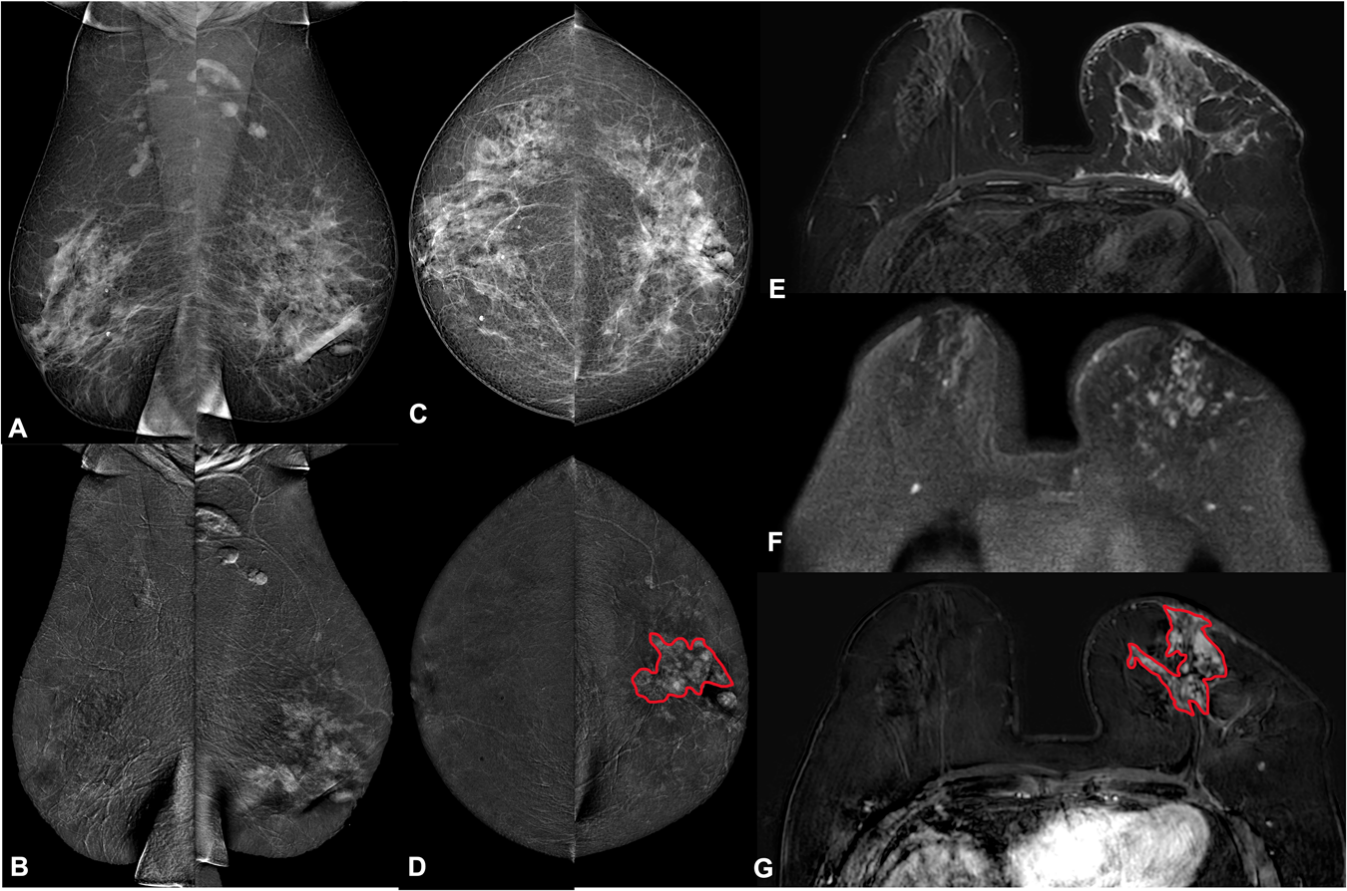
CEM and MRI examinations of a 62-year-old patient with a no-special type (luminal B, G2) multicentric cancer of the left breast with ipsilateral axillary lymph node involvement. **A** Low-energy mediolateral oblique; **B** recombined mediolateral oblique; **C** low-energy craniocaudal; and (**D**) recombined craniocaudal CEM views. **E** Short tau inversion recovery; **F** DWI; and (**G**) post-contrast subtracted axial MRI images. An example of tumor segmentation on recombined CEM and post-contrast subtracted MRI images is illustrated in **D** and **G**, respectively

AI solutions for breast MRI largely remain in the research realm, and their applications range from the detection and classification of lesions to recurrence score and risk assessment, molecular subtyping prediction and treatment response assessment (Fig. 5).

Commercial CADs, available since the early 2000s, offer limited added diagnostic value, especially for experienced radiologists [32]. More recent research models show increased performance. Anaby et al reported AI could detect up to 65% of missed cancers in a retrospective screening dataset [33]. Verburg et al demonstrated an AI model trained on the DENSE trial data dismissed 40% of negatives at a sensitivity of 100% [34], with follow-up validation confirming 33% normal-case dismissal and 46% biopsy reduction [35]. Bhowmik et al, using over 14,000 MRIs to train their model, found 11% of them could be excluded from reading at 100% sensitivity, though specificity remained low (20%) [36]. Jing et al reduced workload by 16% at 98% sensitivity by applying AI to ultrafast sequences (AUC 0.81). The authors developed a model able to automatically pinpoint breast lesions from maximum intensity projection image of the last time-revolved ultrafast sequence, with a significant positive impact on efficiency and diagnostic performance [37]. Oviedo et al’s explainable AI achieved AUC 0.84 in a balanced dataset but dropped to 0.72 in screening-like data [38]. Eppenhof et al reported an AUC of 0.86–0.96 in staging datasets, but only 0.80–0.81 in screening ones [39], highlighting the need for evidence on real-world screening datasets before clinical spread.

AI can predict cancer risk, as proposed by Ha et al, with a CNN able to effectively classify Oncotype DX risk categories (AUC 0.92) [40], and recurrence risk as proposed by Zuo et al [41]. AI models combining MRI with clinical variables can also stratify patients based on their likelihood of developing breast cancer. Saha et al used background parenchymal enhancement (BPE) features to predict cancer onset within 2 years, outperforming radiologists’ subjective assessments [42]. Similarly, Portnoi et al developed a model to assess 5-year breast cancer risk in high-risk women, using a single axial maximum intensity projection, outperforming Tyrer-Cuzick (AUC 0.64 vs. 0.49) [43].

The so-called AI-based “virtual biopsy” on MRI and contrast-enhanced mammography (CEM) can predict molecular subtypes with good performance at a whole-lesion level and per subtype, as demonstrated by several articles including a metanalysis of thirty-five studies: Luminal A (sensitivity: 0.78, specificity: 0.83), Luminal B (sensitivity: 0.75, specificity: 0.68), triple-negative (sensitivity: 0.68, specificity: 0.85) and HER2+ (sensitivity: 0.87, specificity: 0.88) subtypes [44]. Notably, subtype definition differences (e.g., immunohistochemical staining or St. Gallen) may impact accuracy [45].

MRI is key in predicting pathological complete response (pCR) after neoadjuvant treatments [46]. However, identifying non-responders is critical to avoid unnecessary costs associated with ineffective therapies, and several studies have explored AI’s potential to predict response either before or after 1–2 treatment cycles (AUC 0.72–0.93) [47, 48], with improved performance with the addition of clinical data [49]. The integration of longitudinal and multimodal data, including DM, further improves AI performance, as demonstrated by Gao et al [50].

Clinical use of AI systems for breast MRI beyond conventional color coding of dynamic data and image registration cannot yet be recommended. Substantially more research is needed to bring the available algorithms from the research domain toward useful clinical products.

## Contrast-enhanced mammography: preliminary experiences

Relatively limited availability, radiation exposure, potential allergic reactions to iodinated contrast, and reduced specificity compared to MRI are the main limitations of CEM [51]. The development of AI for CEM is still in a very early phase, and further development is required before techniques can even be tested in a clinical environment, potentially assisting in improving its specificity and reducing radiation exposure.

Preliminary research has explored radiomics-based molecular subtype classification on CEM [52–56]. A study on 386 malignant lesions demonstrated that a DL model using low- and high-energy radiomics features predicted Luminal, HER2+, and triple-negative subtypes in the external dataset with AUC of 0.82, 0.83, and 0.68, respectively [56]. Similarly, in a dataset of 367 pathologically confirmed cases, a model integrating high-energy CEM images achieved an AUC of 0.90 for triple negatives [55].

Of note, a recent study has demonstrated how a DL model can create synthetic contrast-enhanced recombined from DM images. Based on a radiologist-based qualitative analysis, synthetic images closely resembled real contrast-enhanced images. Future work will focus on refining DL architectures, external validation, and investigating contrast dose reduction rather than a potential full elimination [57].

## Large language models in breast imaging

Although the evidence is still limited, large language models (LLMs) can impact breast imaging by aiding decision-making, standardizing reporting, and integrating clinical guidelines. Their multimodal capabilities enable text and image analysis, supporting tumor classification and workflow optimization. Despite outperforming humans in some tasks, they require domain-specific training for reliable clinical integration [58, 59]. While some systems may provide accurate information, current clinical use of LLMs without further training needs to be discouraged.

## Summary statement

In breast imaging, several challenges still limit the clinical widespread use of AI algorithms, and even clinically available ones are often not evaluated in real clinical practice. Evidence on the effective use of AI algorithms in mammographic screening is available but is limited on the added diagnostic value for DBT, US and MRI. For all AI applications, real-world trials and long-term impact evaluations are needed, and based on the WHO phases for generating evidence for AI-based medical devices (https://iris.who.int/bitstream/handle/10665/349093/9789240038462-eng.pdf?sequence=1), the status in different modalities is shown in Fig. 1. Based on this, it is not possible to strongly recommend the use of any AI algorithm in breast imaging today. While some AI algorithms may have clinical potential, their use should be regarded as optional. Unless AI is used as a standalone tool for imaging evaluation, the final image interpretation responsibility lies with the radiologist, and available AI tools can only be regarded as a tool to assist the radiologist in this task. As such, it is important to mention that we are of the opinion that radiologists may always overrule AI findings and that there is no need to specify that in the radiological report.

## Patient summary

Artificial intelligence (AI) can have a positive impact on breast cancer care. Asymptomatic women would benefit more from AI tools applied to screening mammography or MRI, symptomatic ones to breast US, and breast cancer patients from those applied to MRI and CEM. The main available evidence is on mammographic screening, where improved diagnostic accuracy and significant reductions in radiologists’ workload have been demonstrated. Most of the commercially available products are in this field and are potentially closer to clinics. The main obstacles to their clinical diffusion are the need for more robust data on their clinical impact and regulatory issues.

## Abbreviations

ABUS: Automated breast ultrasound
AI: Artificial intelligence
ALN: Axillary lymph node
CAD: Computer-aided diagnosis
CEM: Contrast-enhanced mammography
DBT: Digital breast tomosynthesis
DM: Digital mammography
SWE: Shear wave elastography
US: Ultrasound
